# Awareness and Perception of Impacted Wisdom Teeth among Non-Medical Students in Salem: A Cross-Sectional Study

**DOI:** 10.21142/2523-2754-1304-2025-260

**Published:** 2025-11-08

**Authors:** Inisha Shravani, Venkataraman Subhalakshmi, Reena Rachel John

**Affiliations:** 1 Vinayaka Mission’s Sankarachariyar Dental College, Vinayaka Mission’s Research Foundation (VMRF- Deemed to be University). Salem, Tamil Nadu. inishashravani@gmail.com Vinayaka Mission’s Sankarachariyar Dental College Vinayaka Mission’s Research Foundation (VMRF- Deemed to be University) Salem Tamil Nadu inishashravani@gmail.com; 2 Research Associate, Vinayaka Mission’s Sankarachariyar Dental College, Vinayaka Mission’s Research Foundation (VMRF- Deemed to be University). Salem, Tamil Nadu. drvsubhalakshmi@vmsdc.edu.in Research Associate Vinayaka Mission’s Sankarachariyar Dental College Vinayaka Mission’s Research Foundation (VMRF- Deemed to be University) Salem Tamil Nadu drvsubhalakshmi@vmsdc.edu.in; 3 Associate Dean (Research), Vinayaka Mission’s Sankarachariyar Dental College, Vinayaka Mission’s Research Foundation (VMRF- Deemed to be University). Salem, Tamil Nadu. drreenaracheljohn@vmsdc.edu.in Associate Dean (Research) Vinayaka Mission’s Sankarachariyar Dental College Vinayaka Mission’s Research Foundation (VMRF- Deemed to be University) Salem Tamil Nadu drreenaracheljohn@vmsdc.edu.in

**Keywords:** impacted teeth, third molar, surgical removal, awareness, non-medical students, dientes impactados, tercer molar, extracción quirúrgica, concienciación, estudiantes no médicos

## Abstract

**Introduction:**

Impacted teeth may sometimes be asymptomatic but can also be associated with serious complications such as infections, cysts, or tumors. The common symptoms of impacted teeth include pain, swelling, and/or trismus (difficulty opening the mouth). Despite the potential for complications, public awareness of impacted teeth is low. Thus, this research aims to assess the awareness and consequences of impacted teeth among non-medical students.

**Materials and methods:**

This cross-sectional study was conducted among non-medical students in Salem, Tamil Nadu, India. A validated standard questionnaire was prepared in English with multiple-choice and yes or no questions. The survey was conducted online, and the validated questionnaire with an appended informed consent form was sent via Google Form application. The questionnaire consisted of questions about awareness of and the consequences of impacted teeth. The responses of the participants were recorded and analyzed for completeness. Incomplete forms were excluded from the study. Statistical analysis was performed using descriptive statistics, the Chi-square test for categorical variables, and the Mann-Whitney U test for group comparisons. Normality was assessed using the Kolmogorov-Smirnov and Shapiro-Wilk tests.

**Results:**

There were 207 (53.5%) males and 180 (46.5%) females. Of the study participants, 254 participants (65.6%) were unaware of impacted teeth, while 133 (34.4%) reported being aware. Even though females were fewer in number compared to males, they were more aware of the impacted wisdom teeth. Awareness was significantly higher among Arts and Science students compared to Engineering students (Chi-square test, p = 0.032). The study population was primarily between the ages of 17 and 25. The mean age was 20 years. Additionally, about 31% of participants were familiar with the time of eruption of wisdom teeth, indicating a moderate level of awareness among this group regarding a specific type of impacted tooth. This highlights the need for greater educational efforts to increase awareness about impacted teeth in the general population.

**Conclusion:**

The present study indicates that only 34.4% of the non-medical students were aware of wisdom teeth but were unaware of the complications of impacted teeth. Hence, a better awareness of impacted teeth and the associated problems should be created among non-medical students.

## RESUMEN

**Introducción:**

Las piezas dentales impactadas pueden ser asintomáticas en ocasiones, pero también pueden estar asociadas a complicaciones graves como infecciones, quistes o tumores. Los síntomas comunes incluyen dolor, inflamación y/o trismo (dificultad para abrir la boca). A pesar de la posibilidad de complicaciones, la concienciación pública sobre las piezas dentales impactadas es baja. Por lo tanto, esta investigación buscó evaluar la concienciación y las consecuencias de las piezas dentales impactadas entre estudiantes no médicos.

**Materiales y métodos:**

Este estudio transversal se realizó entre estudiantes no médicos en Salem, Tamil Nadu, India. Se elaboró un cuestionario estándar validado en inglés con preguntas de opción múltiple y con las alternativas “sí” o “no”. La encuesta se realizó en línea y el cuestionario validado, junto con un formulario de consentimiento informado adjunto, se envió a través de la aplicación Google Forms. El cuestionario consistió en preguntas sobre la concienciación y las consecuencias de las piezas dentales impactadas. Las respuestas de los participantes se registraron y analizaron para verificar su integridad. Los formularios incompletos fueron excluidos del estudio. El análisis estadístico se realizó mediante estadística descriptiva, la prueba de chi-cuadrado para variables categóricas y la prueba U de Mann-Whitney para las comparaciones de grupos. La normalidad se evaluó mediante las pruebas de Kolmogorov-Smirnov y Shapiro-Wilk.

**Resultados:**

Se incluyeron 207 (53,5%) hombres y 180 (46,5%) mujeres. De los participantes del estudio, 254 (65,6%) desconocían la presencia de dientes impactados, mientras que 133 (34,4%) informaron tenerla. Si bien el número de mujeres fue menor que el de hombres, eran más conscientes de la presencia de muelas del juicio impactadas. El conocimiento fue significativamente mayor entre los estudiantes de Artes y Ciencias que entre los de Ingeniería (prueba de chi-cuadrado, p = 0,032). La población del estudio se encontraba principalmente entre los 17 y los 25 años. La edad media fue de 20 años. Además, aproximadamente el 31% de los participantes conocía el momento de erupción de las muelas del juicio, lo que indica un nivel moderado de conocimiento en este grupo sobre un tipo específico de diente impactado. Esto resalta la necesidad de mayores esfuerzos educativos para aumentar la concienciación sobre los dientes impactados en la población general.

**Conclusión:**

El presente estudio indica que solo el 34,4% de los estudiantes no médicos conocían las muelas del juicio, pero desconocían las complicaciones de los dientes impactados. Por lo tanto, se debe fomentar una mayor concienciación sobre los dientes impactados y los problemas asociados entre los estudiantes no médicos.

## INTRODUCTION

Impaction, as defined by Anderson, is the cessation of the eruption of a tooth caused by a clinically or radiographically detectable physical barrier in the eruption path or by an ectopic position of the tooth ^1^. Archer defines impacted tooth as completely or partially unerupted and positioned against another tooth, bone, or soft tissue, so its further eruption is unlikely ^2^.

The third molars, or wisdom teeth, are the most impacted, followed by maxillary canines and mandibular premolars ^3^^,^^4^. Impacted teeth often remain asymptomatic and may not produce noticeable symptoms, allowing them undetected during routine oral care. However, if left untreated, impacted teeth can lead to various complications, including pericoronitis, dental caries, periodontal disease, root resorption of adjacent teeth, and cyst or tumor formation. To prevent these issues, patients must schedule regular dental checkups. Dentists can identify potential problems early through physical examinations and diagnostic tools like radiographs. Early detection and intervention can help prevent serious complications and improve oral health ^4^^-^^8^. 

Radiographs (such as orthopantomograms), clinical exams, and patient histories are the primary tools for detecting impacted teeth. Early diagnosis and timely surgical intervention can prevent complications. Without appropriate monitoring and management, impacted teeth can lead to facial swelling, trismus, infection, and jaw stiffness, negatively impacting quality of life ^8^^-^^9^. 

Patient awareness and routine dental checkups are essential for identifying and managing impactions. However, medical and dental students' awareness is generally higher than that of their non-medical counterparts. This disparity is concerning given that public knowledge about the risks associated with impacted teeth-such as odontogenic infections, cystic degeneration, or neoplastic changes-remains limited ^10^^-^^12^.

Previous studies have focused on dental or healthcare students, but data on non-medical student populations are sparse. Therefore, assessing knowledge and awareness in this demographic is critical to developing targeted oral health education programs that promote early consultation and intervention. This study uses a structured questionnaire to evaluate the awareness, knowledge, and attitudes regarding impacted teeth among non-medical undergraduate students in Salem City. Thus, the purpose of this study was to identify gaps in awareness and provide recommendations to improve early detection and management through educational outreach.

## MATERIALS AND METHODS

This cross-sectional study was conducted among non-medical undergraduate students from various colleges in Salem, Tamil Nadu, India, following approval from the Vinayaka Mission’s Sankarachariyar Dental College- Institutional Research Committee (IRC No.: IRC/262729022024/S/39).

A standardized, self-administered questionnaire in English was developed to assess the awareness and consequences of impacted teeth. The survey was distributed electronically via Google Forms, with an appended informed consent form. The questionnaire was validated by a panel of 20 Professors and Heads of Departments, each with more than 10 years of teaching and clinical experience, ensuring content validity and relevance.

The questionnaire comprised 14 items, categorized as follows:

• Questions 1-4: Assessed awareness of wisdom teeth
• Questions 5-8: Focused on disturbances or symptoms caused by impacted teeth
• Questions 9-14: Evaluated prior treatment experience and management of wisdom teeth

A total of 387 completed responses were included in the final analysis. Incomplete or partially filled responses were excluded.

### Statistical analysis

The analysis was performed using IBM SPSS Statistics for Windows, Version 22.0 (SPSS Inc., Chicago, IL, USA). Descriptive statistics were applied to calculate frequencies and percentages. The Kolmogorov-Smirnov and Shapiro-Wilk tests were used to assess the normality of the data, and it was found to be non-normally distributed. Chi-square tests were used to compare categorical variables (such as awareness based on age and gender). The Mann-Whitney U test was employed to compare mean knowledge scores between genders and college types. A p-value of ≤ 0.05 was considered statistically significant.

## RESULTS

A total of 387 participants took part in the study, comprising 207 males (53.5%) and 180 females (46.5%). Most participants were between 17 and 25, with a mean age of 20.

Among the respondents, 254 (66%) were unaware of impacted teeth, while 133 (34%) reported awareness. Although there were fewer female participants, their level of awareness was slightly higher than that of males; however, this difference was not statistically significant (Chi-square test, p = 0.513).

Regarding educational background, 233 (60%) of the participants were from Arts and Science colleges, while 114 (30%) were from Engineering colleges. The awareness of impacted wisdom teeth was significantly higher among Arts and Science students than Engineering students (Chi-square test, p = 0.032).

When asked about symptoms related to impacted teeth, 40% of participants reported experiencing pain and swelling, 31% were aware of the typical age of wisdom tooth eruption, and 45% had undergone X-rays or scans based on a dentist’s recommendation.

As shown in Table 2, no significant differences in awareness were observed across age groups (p = 0.456) or gender (p = 0.513). However, a statistically significant difference was noted based on the type of college attended, with students from Arts and Science colleges demonstrating a higher level of awareness than those from Engineering colleges (p = 0.032, Chi-square test).

**Table 1:** Assessment of Study Participants’ Responses to the Questionnaire by Age, Gender, and College Type

Question	18-21	22-24	p-value	Male	Female	p-value	Engineering	Arts & Science	p-value
1.Are you aware of wisdom teeth?									
Yes	97 (25.1%)	30(7.8%)	0.000	77(19.9%)	50(12.9%)	0.031	26 (6.7%)	101(26.1%)	0.001
No	238(61.5%)	22(5.7%)		130(33.6%)	130(33.6%)		95 (24.5%)	165(42.6%)	
2.Do you know which tooth erupts last in the mouth?									
Canine	70 (18.1%)	9 (2.3%)	0.359	47 (12.1%)	32 (8.3%)	0.636	24 (6.2%)	55 (14.2%)	0.042
Incisor	58 (15.0%)	13 (3.4%)		35 (9.0%)	36 (9.3%)		28 (7.2%)	43 (11.1%)	
Premolar	107 (27.6%)	12 (3.1%)		63 (16.3%)	56 (14.5%)		43 (11.1%)	76 (19.6%)	
Wisdom teeth	100 (25.8%)	18 (4.7%)		62 (16.0%)	56 (14.5%)		26 (6.7%)	92 (23.8%)	
3.How many wisdom teeth does a human have?									
2 teeth	90 (23.3%)	9(2.3%)	0.469	62(16.0%)	37 (9.6%)	0.067	34 (8.8%)	65(16.8%)	0.078
3 teeth	122(31.5%)	21(5.4%)		66(17.1%)	77(19.9%)		53 (13.7%)	90(23.3%)	
4 teeth	76 (19.6%)	15(3.9%)		52(13.4%)	39(10.1%)		21 (5.4%)	70(18.1%)	
5 teeth	47 (12.1%)	7 (1.8%)		27 (7.0%)	27 (7.0%)		13 (3.4%)	41(10.6%)	
4.Do you know at what age the wisdom teeth erupt in the oral cavity?									
17-25 yrs	132(34.1%)	23(5.9%)	0.541	93(24.0%)	62(16.0%)	0.050	32(8.3%)	123(31.8%)	0.000
13-16 yrs	154(39.8%)	20(5.2%)		85(22.0%)	89(23.0%)		78(20.2%)	96(24.8%)	
28 -30 years	36(9.3%)	8(2.1%)		19(4.9%)	25(6.5%)		10(2.6%)	34(8.8%)	
35 - 40 years	13(3.4%)	1(0.3%)		10(2.6%)	4(1.0%)		1(0.3%)	13(3.4%)	
5. Do you think it's okay to leave your impacted wisdom teeth untreated?									
Yes	160(41.3%)	19(4.9%)	0.086	104(26.9%)	75(19.4%)	0.050	47(12.1%)	132(34.1%)	0.031
No	175(45.2%)	33(8.5%)		103(26.6%)	105(27.1%)		74(19.1%)	134(34.6%)	
6. Do unerupted (wisdom teeth) or partially erupted teeth affect your routine brushing habits?									
Yes	178(46.0%)	29(7.5%)	0.420	114(29.5%)	93(24.0%)	0.285	67(32.4%)	140(36.2%)	0.348
No	157(40.6%)	23(5.9%)		93(24.0%)	87(22.5%)		54(14.0%)	126(32.6%)	
7. Have you ever experienced pain, swelling or difficulty opening your mouth due to unerupted teeth?									
Yes	191(49.4%)	26(6.7%)	0.212	112(28.9%)	105(27.1%)	0.232	67(17.3%)	150(38.8%)	0.469
No	144(37.2%)	26(6.7%)		95(24.5%)	75(19.4%)		54(14.0%)	116(30.0%)	
8. Have you experienced any of the following problems due to improperly or partially erupted impacted teeth or wisdom teeth?									
Cheek biting	114(29.5%)	15(3.9%)	0.068	73(18.9%)	56(14.5%)	0.001	41(10.6%)	88(22.7%)	0.448
Pain	111(28.7%)	25(6.5%)		87(22.5%)	49(12.7%)		38(9.8%)	98(25.3%)	
Difficulty in mouth opening	52(13.4%)	9(2.3%)		23(5.9%)	38(9.8%)		24(6.2%)	37(9.6%)	
Swelling	58(15.0%)	3(0.8%)		24(6.2%)	37(9.6%)		18(4.7%)	43(11.1%)	
9. What was your first treatment of choice for problems related to unerupted (or) wisdom teeth?	58(15.0%)	3(0.8%)	0.066	24(6.2%)	37(9.6%)	0.003	18(4.7%)	43(11.1%)	
Self medication	132(34.1%)	22(5.7%)		85(22.0%)	69(17.8%)		18(4.7%)	43(11.1%)	
General dentist	101(26.1%)	14(3.6%)		56(14.5%)	59(15.2%)		57(14.7%)	97(25.1%)	
General Physician	35(9.0%)	11(2.8%)		35(9.0%)	11(2.8%)		38(9.8%)	77(19.9%)	
Consulting a dental specialist	67(17.3%)	5(1.3%)		31(8.0%)	41(10.6%)		9(2.3%)	37(9.6%)	
10.Have you ever visited a dentist for unerupted or wisdom teeth?( If the answer is no then skip the 11th question)									
yes	166(42.9%)	34(8.8%)	0.023	103(26.6%)	97(25.1%)	0.239	17(4.4%)	55(14.2%)	0.333
no	169(43.7%)	18(4.7%)		104(26.9%)	83(21.4%)		65(16.8%)	135(34.9%)	
11.If 1,What was the treatment done for the unerupted or wisdom teeth?									
no	164(42.4%)	17(4.4%)	0.015	101(26.1%)	80((20.7%)	0.023	56(14.5%)	131(33.9%)	0.045
Extraction	84(21.7%)	11(2.8%)		39(10.1%)	56(14.5%)		53(13.7%)	128(33.1%)	
Medications prescribed	66(17.1%)	16(4.1%)		47(12.1%)	35(9.0%)		23(5.9%)	72(18.6%)	
Removal of tissue	21(5.4%)	8(2.1%)		20(5.2%)	9(2.3%)		31(8.0%)	51(13.2%)	
12.Have you had any x rays/scans taken for unerupted or wisdom teeth before?									
yes	151(39.0%)	23(5.9)	0.516	86(22.2)	88(22.7)	0.089	14(3.6)	15(3.9)	0.509
no	184(47.5%)	29(7.5)		121(31.3)	92(23.8)		54(14.0)	120(31.0)	
13.Do you know that cysts/tumours can arise from unerupted or partially erupted wisdom teeth?									
yes	47(12.1%)	19(4.9%)	0.000	49(12.7%)	17(4.4%)	0.000	67(17.3%)	146(37.7%)	0.000
no	288(74.4)	33(8.5%)		158(40.8%)	163(42.1%)		5(1.3%)	61(15.8%)	
14.Have you undergone any surgical procedures related to unerupted or wisdom teeth?									
yes	123(31.8%)	26(6.7%)	0.048	81(20.9%)	68(17.6%)	0.434	116(30.0%)	205(53.8%)	0.126
no	212(54.8%)	26(6.7%)		126(32.6)	112(28.9%)		41(10.6%)	108(27.9%)	

Chi-square test

**Table 2:** Comparison of the levels of awareness based on demographic variables

Variables	Low	High	P value
	N (%)	N (%)	
Age group			
18-21	301(77.8)	34(8.8)	0.456
21-24	46(11.9)	6(1.6)	
Gender			
Male	186(48.1)	21(5.4)	0.513
Female	161(41.6)	19(4.9)	
College type			
Engineering	114(29.5)	7(1.8)	0.032
Arts & Science	233(60.2)	33(8.5)	

Chi-square tests

## DISCUSSION

Various studies on impaction show that tooth impaction occurs in 6% to 19% of cases, with many studies showing that impaction is more prevalent in females than in males. The primary causes of eruption failure include insufficient arch length and inadequate space for proper eruption ^10^^-^^13^.

The mandibular third molar, commonly known as the wisdom tooth, is the most frequently affected by impaction, followed by the maxillary third molar, maxillary canine, mandibular premolars, maxillary premolars, and second molars. The teeth with the lowest impaction rates are the mandibular incisors, first molars, and primary teeth. Syndromes like Gardner syndrome, nevoid basal cell carcinoma syndrome (Gorlin syndrome), and cleidocranial dysostosis have been linked to multiple dental impactions. Also, hormonal imbalances and other metabolic disorders can contribute to the development of impacted teeth. Impacted teeth lead to a range of complications, including the formation of cysts or tumors, dental decay, root resorption, and periodontal disease ^14^.

The age at which third molars begin to show signs and symptoms can vary widely, influenced by genetics, gender, and other environmental influences ^12^^,^^14^.

Our research was conducted among non-medical students aged 17 to 25 in Salem. The findings indicated that just 34% were aware of impacted teeth. Although most participants were male, female students showed slightly higher awareness levels, though this difference was not statistically significant (p = 0.513). Notably, arts and science students had a significantly greater awareness than those in Engineering (p = 0.032). This may be attributed to broader exposure to general education subjects or better engagement with public health topics.

Only 31% of participants recognized that wisdom teeth are the last to emerge, and 40% were aware of the usual age for their eruption. While 45% reported having had radiographic evaluations, a large portion (83%) did not know about potential complications like cysts, cavities, gum disease, or pericoronitis. These statistics highlight a significant knowledge gap and the necessity for early educational initiatives.

Regarding symptoms, 40% of participants reported experiencing pain and swelling, while others mentioned issues like cheek biting, difficulty opening their mouths, and swelling. Despite these symptoms, many opted for self-medication (40%) instead of seeking professional dental care. Only 30% consulted a dentist, and 39% had undergone minor surgical procedures. These patterns indicate a lack of proactive treatment-seeking behavior, suggesting obstacles such as low awareness, fear, or financial concerns.

Previous studies support these findings. For instance, Sun et al. reported low awareness among non-medical students in China, with nearly 50% demonstrating poor knowledge.^15^ Similarly, Özbay et al. ^9^ observed moderate awareness in a Turkish population, and Balakrishnan et al. found that even dental students had limited knowledge during their preclinical years ^16^.

Early diagnosis through radiographs and regular check-ups should be emphasized. Dentigerous cysts, root resorption, and other serious complications often develop silently. Educational initiatives must aim to bridge this awareness gap, especially in populations outside the health sciences.

Balakrishnan et al. ^16^ in 2020 conducted a study among dental students and found that 50% of the participants knew impacted teeth. In our research, non-medical students demonstrated a lower level of knowledge about impacted teeth. This difference is due to the potential influence of specialized education on awareness levels.

Another study conducted by Twayna et al. ^17^^)^ among dental students revealed that before joining the Bachelor in Dental Surgery (BDS) course, only 56% had prior knowledge of "impacted teeth". However, the study showed that a majority of students (81%) were familiar with third molar impactions, 43% (62 students) were aware of the types of impactions, and 69% (100 students) knew about the complications associated with not removing the impacted teeth. This implies that dental education enhances awareness and understanding of impacted teeth ^17^.

Our findings reinforce the need for accessible and engaging educational content about oral health. Leveraging social media platforms and integrating dental awareness modules into general education curricula may enhance public understanding and encourage healthier behaviour.

Overall, the results emphasize a pressing need for oral health promotion strategies for young adults in academic settings, particularly non-medical streams.

This study is limited by its cross-sectional design and reliance on self-reported data, which may introduce recall bias.

## CONCLUSION

In the current study, only 34% of non-medical participants were aware of impacted wisdom teeth and their implications for oral health. Therefore, the widespread influence of social media, which offers an excellent platform to raise awareness among the younger generation, can be a useful tool. Educational awareness on proper brushing, risks of impaction, and when complications arise, the surgical removal of the affected impacted tooth should be concentrated on the general public. The public can also be encouraged to regular dental visits through social media and other platforms, promoting early intervention and better oral health among young people.
